# Male Tuberous Breast: A Rare Variant of Gynecomastia

**DOI:** 10.7759/cureus.107219

**Published:** 2026-04-17

**Authors:** Anas Ankiz, Soufyane El Kadiri, Doha Arreyouchi, Ayat Allah Oufkir

**Affiliations:** 1 Department of Plastic, Reconstructive and Burn Surgery, Mohammed VI University Hospital, Oujda, MAR; 2 Department of Plastic and Reconstructive Surgery, Faculty of Medicine and Pharmacy, Mohammed First University, Oujda, MAR; 3 Department of Plastic and Reconstructive Surgery, Mohammed VI University Hospital, Oujda, MAR; 4 Department of Plastic Surgery, Faculty of Medicine and Pharmacy, Mohammed First University, Oujda, MAR

**Keywords:** breast base constriction, gynecomastia, male tuberous breast, nipple-areolar complex, surgical correction

## Abstract

Tuberous breast deformity is well described in females but remains rare in males, where it represents an uncommon morphological variant of gynecomastia. It is characterized by a constricted breast base, enlarged nipple-areolar complex (NAC), inframammary fold indentation, and apparent parenchymal herniation into the areola. We report the case of a 17-year-old male presenting with progressive bilateral breast enlargement since the age of 11, with marked worsening at 16 years and significant psychosocial distress. Clinical examination revealed bilateral glandular gynecomastia with enlarged NACs, mild asymmetry, and global base constriction consistent with a severe tuberous deformity. Ultrasonography showed homogeneous glandular hypertrophy, and endocrine evaluation was normal. Surgical correction using glandular transection and release of constricting elements resulted in satisfactory aesthetic outcomes without complications. Male tuberous breast deformity is an underrecognized entity requiring tailored surgical management addressing both glandular excess and base constriction.

## Introduction

Gynecomastia is a benign enlargement of the male breast and is a frequent source of embarrassment and low self-esteem, particularly in adolescence [1,2]. Although typical presentations are commonly classified according to Simon et al. [3], certain variants display morphologic features resembling the female tuberous breast deformity. The tuberous breast was first described by Rees and Aston in 1976 [4]. The male tuberous breast (MTB) is a rare and underreported condition characterized by a constricting fibrous component creating an inframammary fold (IMF)-like imprint, a narrowed breast base, enlargement of the nipple-areolar complex (NAC), and a feminized projection of the breast mound [5,6]. Because the IMF imprint tends to persist due to its structural “memory,” correction can be challenging and may require targeted release and remodeling rather than simple gland excision alone [5,7]. Despite its benign nature, however, MTB is clinically important as it may be misdiagnosed as typical gynecomastia, leading to inappropriate management. In addition, it may have a significant psychosocial impact, particularly in adolescent patients, and requires specific surgical considerations due to its unique anatomical features. We report a case of bilateral MTB in an adolescent male and provide a focused review of the literature.

## Case presentation

**Figure 1:**
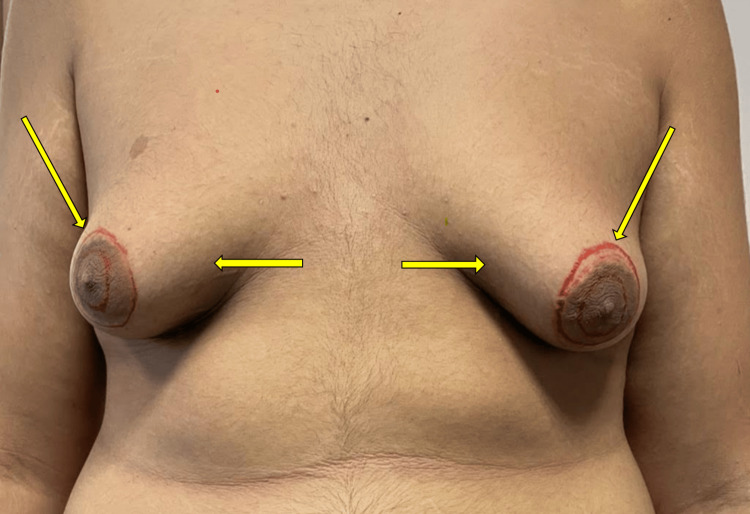
Preoperative view showing bilateral tuberous breast deformity. Preoperative surgical markings outlining the nipple-areolar complexes are visible. Arrows indicate areolar enlargement and breast base constriction. Written informed consent was obtained from the patient for publication of this case report and accompanying images.

**Figure 2:**
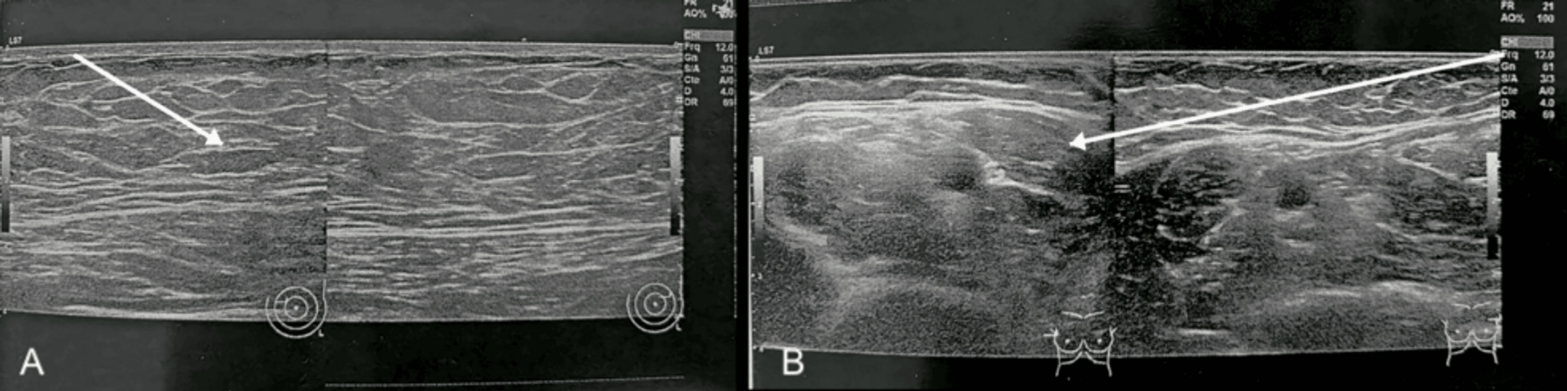
(A-B) Bilateral breast ultrasonography. (A-B) Arrows indicate retroareolar homogeneous glandular hypertrophy without focal lesions.

**Figure 3:**
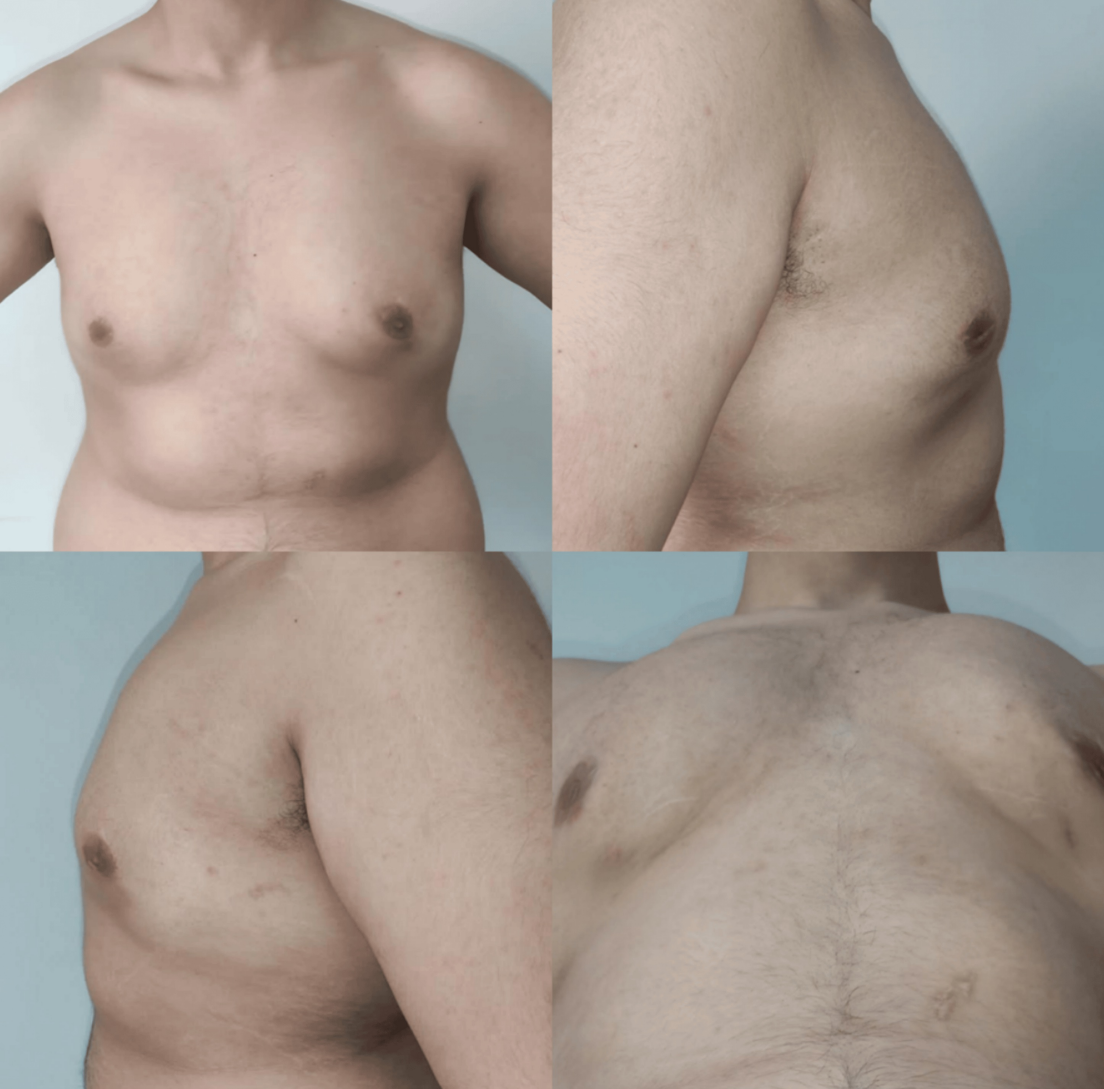
Postoperative views at three-month follow-up. Frontal, right lateral, left lateral, and inferior views demonstrate improved chest contour and correction of the tuberous deformity. Written informed consent was obtained from the patient for publication of this case report and accompanying images.

A 17-year-old male was referred to our department for progressive bilateral breast enlargement evolving since the age of 11, with marked worsening at 16 years and significant psychosocial distress. His past medical history included an undocumented cervical adenectomy at the age of four. The family history was notable for bilateral gynecomastia in his younger brother. On preoperative examination, the patient appeared his stated age, with a body mass index of 31 kg/m^2^. Clinical evaluation demonstrated bilateral glandular gynecomastia with enlarged NACs measuring 2.5 cm in diameter, mild breast asymmetry, and global base deficiency consistent with a severe tuberous deformity (von Heimburg type III/IV equivalent) based on marked base constriction, widened areola, and significant breast projection (Figure 1). No axillary masses, nipple retraction, or nipple discharge were observed. Marked abdominal skin excess with striae was also noted. Bilateral breast ultrasonography revealed homogeneous glandular hypertrophy without nodular or cystic lesions (Figure 2). Endocrine evaluation, including serum testosterone, estradiol, luteinizing hormone (LH), follicle-stimulating hormone (FSH), prolactin, and thyroid-stimulating hormone (TSH), was within normal limits, thereby excluding secondary causes of gynecomastia. Surgical correction was performed using an anterior approach based on glandular transection, according to the principles described by Ribeiro [8]. The procedure included release of the constricting components, with radial scoring and redistribution of glandular tissue to expand the constricted base and improve contour, thereby correcting the tuberous morphology and restoring a masculine thoracic contour. Particular attention was paid to releasing the constricted breast base to prevent persistence of the IMF imprint. A compressive dressing was applied at the conclusion of the procedure, and a thoracic compression garment was prescribed. The postoperative course was uneventful. At three months follow-up, no complications were detected, and the patient expressed satisfaction with the aesthetic outcome. A longer follow-up is ongoing to assess the stability of the result (Figure 3).

## Discussion

MTB represents a rare morphological variant of gynecomastia. While tuberous breast deformity has been described in females since the work of Rees and Aston, its occurrence in males remains uncommon and underreported [4,6,7], and its true incidence remains unknown [7].

Classical gynecomastia is often categorized using the Simon classification [3]. However, MTB does not fit well within this framework, as it combines base constriction, enlarged NAC, IMF imprint, and apparent areolar/parenchymal herniation, often with disproportionate skin excess [5].

The largest clinical series, including 21 patients, reported predominantly bilateral and symmetrical deformities, frequently associated with an overweight status. The authors highlighted the persistence of the IMF imprint due to its structural “memory,” which complicates surgical correction [7].

The pathophysiology remains debated. Proposed mechanisms include a constricting fascial ring limiting radial expansion, abnormal dermofascial adherence, and genetically influenced collagen abnormalities. Familial occurrence, as observed in our patient, further supports a possible hereditary component [6,9-11].

Classification systems developed for female tuberous breasts are often applied to males. Von Heimburg described four types based on lower pole hypoplasia and base constriction [12], later modified by Grolleau et al. [9,13]. However, no classification system has been specifically validated for male patients.

Surgical correction aims to restore a masculine chest contour by addressing base constriction, NAC abnormalities, and skin excess [6]. Techniques vary according to severity. Previous reports have described approaches ranging from circumareolar reduction to Wise-pattern reduction with free nipple grafting in severe cases, although aesthetic limitations such as hypopigmentation have been noted [5,6]. More recent series emphasize targeted release of the constricting IMF component with parenchymal remodeling, often combined with liposuction, achieving high satisfaction rates and low complication rates [7].

In the present case, an anterior approach with glandular transection and release of constricting elements was used to correct base constriction and IMF imprint while preserving NAC vascularity and minimizing scarring. Recognizing MTB as a distinct entity is essential, as mismanagement of simple gynecomastia may lead to suboptimal outcomes, including persistent IMF indentation and areolar bulging. Given the significant psychosocial impact of chest deformities in adolescents, achieving a stable masculine thoracic contour is of considerable clinical importance [2].

## Conclusions

MTB deformity is a rare and distinct clinical entity that may be misdiagnosed as typical gynecomastia. Accurate recognition and individualized surgical planning are essential for optimal management. Surgical techniques addressing both glandular excess and base constriction, including the IMF component, may achieve satisfactory aesthetic outcomes, as illustrated in this case, with acceptable morbidity. These findings are based on a single case with short-term follow-up and should be interpreted with caution.
